# powerEQTL: an R package and shiny application for sample size and power calculation of bulk tissue and single-cell eQTL analysis

**DOI:** 10.1093/bioinformatics/btab385

**Published:** 2021-05-19

**Authors:** Xianjun Dong, Xiaoqi Li, Tzuu-Wang Chang, Clemens R Scherzer, Scott T Weiss, Weiliang Qiu

**Affiliations:** Genomics and Bioinformatics Hub, Department of Neurology, Brigham and Women’s Hospital, Harvard Medical School, Boston, MA 02115, USA; Center for Advanced Parkinson Research and Precision Neurology Program, Department of Neurology, Brigham and Women’s Hospital, Harvard Medical School, Boston, MA 02115, USA; Aligning Science Across Parkinson’s (ASAP) Collaborative Research Network, Chevy Chase, MD 20815, USA; Genomics and Bioinformatics Hub, Department of Neurology, Brigham and Women’s Hospital, Harvard Medical School, Boston, MA 02115, USA; Molecular Pathological Epidemiology Program, Department of Pathology, Brigham and Women’s Hospital, Harvard Medical School, Boston, MA 02115, USA; Center for Advanced Parkinson Research and Precision Neurology Program, Department of Neurology, Brigham and Women’s Hospital, Harvard Medical School, Boston, MA 02115, USA; Aligning Science Across Parkinson’s (ASAP) Collaborative Research Network, Chevy Chase, MD 20815, USA; Channing Division of Network Medicine, Department of Medicine, Brigham and Women’s Hospital, Harvard Medical School, Boston, MA 02115, USA; Non-Clinical Efficacy & Safety, Biostatistics & Programming Department, Sanofi, Framingham, MA 01701, USA

## Abstract

**Summary:**

Genome-wide association studies (GWAS) have revealed thousands of genetic loci for common diseases. One of the main challenges in the post-GWAS era is to understand the causality of the genetic variants. Expression quantitative trait locus (eQTL) analysis is an effective way to address this question by examining the relationship between gene expression and genetic variation in a sufficiently powered cohort. However, it is frequently a challenge to determine the sample size at which a variant with a specific allele frequency will be detected to associate with gene expression with sufficient power. This is a particularly difficult task for single-cell RNAseq studies. Therefore, a user-friendly tool to estimate statistical power for eQTL analyses in both bulk tissue and single-cell data is needed. Here, we presented an R package called powerEQTL with flexible functions to estimate power, minimal sample size or detectable minor allele frequency for both bulk tissue and single-cell eQTL analysis. A user-friendly, program-free web application is also provided, allowing users to calculate and visualize the parameters interactively.

**Availability and implementation:**

The powerEQTL R package source code and online tutorial are freely available at CRAN: https://cran.r-project.org/web/packages/powerEQTL/. The R shiny application is publicly hosted at https://bwhbioinfo.shinyapps.io/powerEQTL/.

**Supplementary information:**

Supplementary data are available at *Bioinformatics* online.

## 1 Introduction

Genome-wide association studies (GWAS) have revealed genetic risk loci for thousands of traits or diseases (Buniello *et al.*, 2019; MacArthur *et al.*, 2017). Nearly 90% of the GWAS loci are located in non-coding regions (Edwards *et al.*, 2013), suggesting that they may play a role by influencing gene expression. One of the main challenges in the post-GWAS era is understanding how these genetic variants cause the phenotype, for example, by regulating the expression of disease-associated or tissue-specific genes. Expression quantitative trait locus (eQTL) analysis has provided such a framework to test the effect of genetic variation on gene expression (Nica and Dermitzakis, 2013). For instance, the Genotype-Tissue Expression (GTEx) project has performed eQTL analysis between genetic variation and genome-wide gene expression in 54 non-diseased tissue sites across nearly 1000 individuals, providing a comprehensive public resource to understand the effect of genetic variants in a wide spectrum of tissue bank samples (GTEx Consortium, 2013, 2015). Enhancing GTEx (eGTEx) further extended this effort to include more intermediate molecular phenotypes other than gene expression (eGTEx Project, 2017). Recent increases in single-cell genomics will allow mapping eQTLs across different cell types, in dynamic processes and in 3D spaces, many of which are obscured when using bulk methods (van der Wijst *et al.*, 2018, 2020). One of the critical steps common to all eQTL experiments is to determine the minimum sample size with enough power to detect variants with a low frequency (e.g. minor allele frequency less than 5%) but a substantial effect on gene expression. However, there is no such tool available for sample size and power calculation for eQTL analysis.

Here, we developed equation-based statistical models to calculate sample size and power for an eQTL analysis in both bulk tissue and single-cell settings. The tool, called powerEQTL, was implemented in both an R package and an interactive online application.

## 2 Materials and methods

### 2.1 Bulk tissue eQTL

Bulk tissue eQTL is to identify the downstream effects of disease-associated genetic variants on the gene expression measured at the bulk tissue level. Because of the affordable price (compared to a single-cell experiment) and the convenience to get enough volume of RNAs from bulk tissue, bulk RNA-sequencing is still the most widely used technique to profile the transcriptome of a tissue nowadays. Gene expression values were quantified on tissue homogenates, usually one sample per subject, for a number of subjects. Normalized gene expressions were then compared among groups of subjects with different genotypes. Since the effect sizes of eQTL are usually small and the large number of gene-SNP pairs leads to a multiple-testing issue (Huang *et al.*, 2018), a proper power analysis including sample size and power calculation is needed before performing experiments.

We implemented the power analysis of bulk tissue eQTL based on two different models, one-way unbalanced ANOVA and simple linear regression (see Online Supplementary Document). They both test for the potential association between genotype and gene expression. The difference lies in that ANOVA test treats the genotype as a categorical data (e.g. AA, AB and BB) and tests the potential non-linear association, while simple linear regression treats genotype as continuous variable using additive coding (e.g. 0 for AA, 1 for AB and 2 for BB, where B is the minor allele) and tests the linear association. GTEx project used the one-way unbalanced ANOVA model in their analysis (GTEx Consortium, 2013). We implemented the two models in functions of *powerEQTL.ANOVA* and *powerEQTL.SLR* in our R package, respectively. Note that if we know the association is linear, *powerEQTL.SLR* would be more powerful than *powerEQTL.ANOVA*. This is because categorizing a continuous-type variable to a set of nominal-type variables would lose information.

Since type I error rate (*α*), type II error rate (*β* or 1-power), effect size and sample size are interrelated in power analysis, we could calculate any one of them if we know the remaining three. We implemented functions to allow calculating any one of these four parameters (power, sample size, slope and minimum allowable MAF) by setting the corresponding parameter as NULL and providing values for the other three parameters in *powerEQTL.SLR*.

### 2.2 Single-cell eQTL

Unlike bulk tissue RNAseq, single-cell RNAseq usually profiles thousands of cells per sample, which provides a better representation for the gene expression distribution of a tissue than a single value from bulk RNAseq. However, the gene expressions among cells within a sample are not independent, e.g. cells from one tissue sample are assumed more correlated than cells between samples. The structured data requires a different model for power analysis.

In this study, we implemented two ways to compute the power of single-cell eQTL (sc-eQTL) analysis. First, we modeled the association of genotype to pre-processed single-cell RNA expression by using a linear mixed effects model: *y*_ij_ = *β*_0i_ + *β*_1_ ∗ *x*_i_ + *ε*_ij_, where *y*_ij_ is the gene expression level for the *j*th cell of the *i*th subject, *x*_i_ is the genotype for the *i*th subject using additive coding (e.g. 0, 1 and 2). The random intercept *β*_0i_ and error term *ε*_ij_ are normally distributed (see Online Supplementary Document for details). The power to test if the slope *β*_1_ is different from zero is implemented in the function *powerEQTL.scRNAseq* with parameters of subject size (*n*), number of cells per subject (*m*), slope (*β*_1_), standard deviation of the gene expression (*σ_y_*), MAF, intra-subject correlation (i.e. correlation between *y*_ij_ and *y*_ik_ for the *j*th and *k*th cells of the *i*th subject, *ρ*), and number of SNP-gene pairs (*nTest*). Similarly, the function can be used to calculate one of the four parameters (power, sample size, minimum detectable slope and minimum allowable MAF) by setting the corresponding parameter as NULL and providing values for the other three parameters.

Second, we directly modeled the read counts of genes by zero-inflated negative binomial (ZINB) distribution to account for the excess of zeros in single-cell RNAseq data. We provided the function *powerEQTL_scRNAseq.sim* to implement a simulation-based power calculation for sc-eQTL based on a ZINB mixed-effects model. To alleviate the intense computation of simulation studies, *powerEQTL_scRNAseq.sim* provides parallel computing capacity.

## 3 Result

**Fig. 1.**
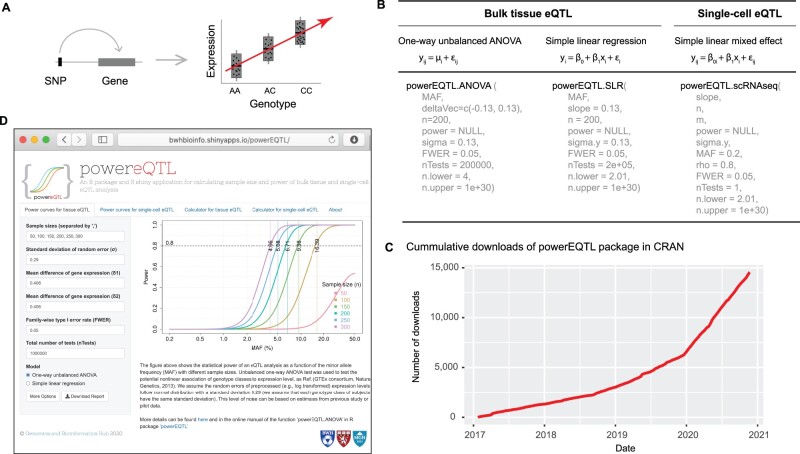
(**A**) eQTL schema. (**B**) The main models and functions in the powerEQTL package. (**C**) Downloads summary of powerEQTL since its original repository on CRAN (data generated by cranlog R package). (**D**) Screenshot of powerEQTL R shiny application

The powerEQTL R package is available in CRAN and has been downloaded over 10 000 times since its first deployment(see Fig. 1). We also implemented the functions for power and sample size calculation in an online, interactive, program-free web application using R Shiny. Power curves of different MAFs for multiple sample sizes are visualized and downloadable for both bulk tissue and sc-eQTL. The calculator pages allow users to freely play with the parameters for tissue and sc-eQTL power analysis. The default values for parameters are based on the parameters from the GTEx cohort [see the ‘Power analysis’ section in (GTEx Consortium, 2013)]. We recommend that users extrapolate their own parameters from pertinent pilot data or appropriate public datasets. This package also has limitations. Covariates such as sex, age and disease traits may influence eQTL relationships and are not accounted for in this model. Moreover, it is conceivable that some eQTLs are not well captured by simple linear or categorical models.

## 4 Discussion

While several R or Bioconductor packages are available for omics sample size and power calculation, such as sizepower (equation-based, 2006), RNASeqPower (equation-based, 2013), PROPER (simulation-based, 2015), powsimR (simulation-based, 2017), RnaSeqSampleSize (2018), ssizeRNA (equation-based, 2019), PowerSampleSize, pwrEWAS and powerGWASinteraction, we are not aware of a package specifically for eQTL power analysis. To apply powerEQTL to RNAseq data, appropriate data transformation is needed to convert counts to continuous data, such as voom (Law *et al.*, 2014), countTransformers (Zhang *et al.*, 2019) or data aggregation (e.g. taking the sum, median or mean expression levels across cells/nuclei from each sample) with appropriate transformations (Cuomo *et al.*, 2020, 2021; Jerber *et al.*, 2021; van der Wijst *et al.*, 2018). In addition to scRNAseq, other structured data, such as scATACseq, single-cell methylation, grouped cell lines etc. can also be applied to this eQTL model. Adding a random effect to account for variable number of cells has been shown to improve eQTL discovery power (Jerber *et al.*, 2021). However, it would be a challenge to calculate power at design stage to incorporate numbers of cells since the numbers of cells would not be known until the user finishes data collection. A future extension to the powerEQTL package/shiny app is to incorporate the information about kinship matrix and variations of number of cells/reads among subjects for power calculation of sc-eQTL.

## Supplementary Material

btab385_Supplementary_Data
