# Supplementary material for: powerEQTL: an R package and shiny application for sample size and power calculation of bulk tissue and single-cell eQTL analysis
Source: Bioinformatics. 2021 May 19;37(22):4269–71. doi: 10.1093/bioinformatics/btab385 (PMC9492284; doi:10.1093/bioinformatics/btab385)
Supplement: btab385_Supplementary_Data [file btab385_supplementary_data.pdf]

Online Supplementary Document for "powerEQTL: An R package and shiny application for sample size and power calculation of bulk tissue and single-cell eQTL analysis"

Xianjun Dong[1,2,3*], Xiaoqi Li[1*], Tzuu-Wang Chang[4], Clemens R. Scherzer[2,3], Scott T. Weiss[5], Weiliang Qiu[6]

[1]: Genomics and Bioinformatics Hub, Brigham and Women's Hospital, Harvard Medical School, Boston, MA 02115

[2]: Center for Advanced Parkinson Research and Precision Neurology Program, Brigham and Women's Hospital, Harvard Medical School, Boston, MA 02115

[3]: Aligning Science Across Parkinsons (ASAP) Collaborative Research Network, Chevy Chase, MD, 20815

[4]: Molecular Pathological Epidemiology Program, Brigham and Women's Hospital, Harvard Medical School, Boston, MA 02115

[5]: Channing Division of Network Medicine, Brigham and Women's Hospital, Harvard Medical School, Boston, MA 02115

[6]: Non-Clinical Efficacy & Safety, Biostatistics & Programming, Sanofi, Framingham, MA 01701

*: These authors contributed equally.

Contact: Xianjun Dong (xdong@rics.bwh.harvard.edu), Weiliang Qiu (weiliang.qiu@sanofi.com)

Contents

1 Power Calculation for Bulk Tissue eQTL Based on ANOVA **3**

1.1 Introduction 3

1.2 General formula 3
1.3 Simplification of non-central parameter 5

2 Power Calculation for Bulk Tissue eQTL Based on SLR **7**
2.1 Introduction 7
2.2 Mean and variance of additive coded genotype 8
2.2.1 Hardy-Weinberg equilibrium 8
2.3 Power Calculation of eQTL based on simple linear regression 9
2.3.1 Power calculation for simple linear regression 9

3 Power Calculation for Single-Cell eQTL **12**
3.1 A Linear Mixed Effects Model 12
3.2 Hypotheses 13
3.3 Power calculation formula 14
3.4 Discussion 14

4 Power Calculation for Single-Cell eQTL via Simulation based on ZINB Mixed Effects Regression Model **15**
4.1 ZINB mixed effects regression model 15
4.2 Power calculation via simulation 16
4.3 Parameter estimates for ZINB mixed effects regression model 16
4.4 Parallel computing 17

A Vector representation **18**

B Generalized least squares estimate when variance-covariance matrix is known **20**

C Mean, variance, and distribution of the generalized least squares estimate **21**

D Calculating the power for testing if the slope is equal to zero **21**

E Calculation of the variance of the slope estimate **23**

F Power calculation formula revisit **27**

G Variance of genotype under Hardy-Weinberg Equilibrium **27**

1 Power Calculation for Bulk Tissue eQTL Based on ANOVA

1.1 Introduction

If we would like to test potential non-linear relationship between genotype of a SNP and expression of a gene, we can use un-balanced one-way ANOVA. Actually, an article published by the GTEx Consortium in 2013[3] used this approach.

1.2 General formula

Suppose there are $k = 3$ groups of subjects: (1) mutation homozygotes; (2) heterozygotes; and (3) wildtype homozygotes. We would like to test if the mean expression μ_i, $i = 1, \ldots, k$, of the gene is the same among the k groups of subjects. We can use the following one-way ANOVA model to characterize the relationship between observed gene expression level y_{ij} and the population mean expression level μ_i:

$$\begin{aligned} y_{ij} =& \mu_i + \epsilon_{ij}, \quad \epsilon_{ij} \sim N\left(0, \sigma^2\right), \\ i =& 1, \ldots, k, \\ j =& 1, \ldots, n_i, \end{aligned} \tag{1}$$

where y_{ij} is the observed gene expression level for the j-th subject in the i-th group, μ_i is the mean gene expression level of the i-th group, ϵ_{ij} is the random error, k is the number of groups, n_i is the number of subjects in the i-th group. Denote the total number of subjects as $N = \sum_{i=1}^k n_i$. That is, we have n_1 mutation homozygotes, n_2 heterozygotes, and n_3 wildtype homozygotes.

We would like to test the null hypothesis H_0 and alternative hypothesis H_1:

$$\begin{aligned} &H_0 : \mu_1 = \mu_2 = \mu_3, \\ &H_1 : \text{not all } \mu_i \text{ are the same.} \end{aligned} \tag{2}$$

It is well know that the test statistic for Hypotheses (2) is the F statistic

$$F = \frac{MS_{grp}}{MSE}, \tag{3}$$

where MS_{grp} is the mean square of group effect and MSE is the mean square of error.

We will reject the null hypothesis H_0 if the test statistic F is large enough. The type I error rate α is defined as

$$\alpha = Pr\left(F > c|H_0\right) = 1 - Pr\left(F < c|H_0\right), \tag{4}$$

where c is the rejection boundary (i.e., cutoff).

Under H_0, the test statistic F follows the F distribution with degrees of freedom $df_1 = k-1$ and $df_2 = N-k$ (Denote it as $F_{k-1,N-k}$). Hence, the cutoff c is the upper 100α percentile of the F distribution $F_{k-1,N-k}$. Denote

$$c = F_{1-\alpha}\left(k-1, N-k\right). \tag{5}$$

Under H_1, the test statistic F follows the non-central F distribution with degrees of freedom $df_1 = k-1$, $df_2 = N-k$, and non-centrality parameter λ (Denote it as $F_{k-1,N-k,\lambda}$).

According to OBrien and Muller (1993)[2], the non-central parameter λ is defined as

$$\lambda = \frac{N}{\sigma^2}\sum_{i=1}^{k}\omega_i\left(\mu_i - \mu\right)^2, \tag{6}$$

where μ_i is the mean value for the i-th level, $\omega_i = n_i/N$ is the weight for the i-th level, and μ is the overall mean,

$$\mu = \sum_{i=1}^{k}\omega_i\mu_i. \tag{7}$$

Hence, the power calculation formula for un-balanced one-way ANOVA with k levels is

$$\begin{aligned} power =& Pr\left(F > c|H_1\right) \\ =& Pr\left(F \geq F_{1-\alpha}\left(k-1, N-k\right) \middle| F \sim F_{k-1,N-k,\lambda}\right)\right). \end{aligned} \tag{8}$$

1.3 Simplification of non-central parameter

For our case, $k = 3$ and the non-centrality parameter λ can be rewritten as

$$\lambda = \frac{N}{\sigma^2} \sum_{i=1}^{3} w_i \left(\mu_i - \mu\right)^2. \tag{9}$$

By assuming Hardy-Weinberg Equilibrium, we have

$$\begin{aligned}
w_1 &= \frac{n_1}{N} = \theta^2 \text{ (genotype frequency for mutation homozygotes)},\\
w_2 &= \frac{n_2}{N} = 2\theta(1-\theta) \text{ (genotype frequency for heterozygotes)},\\
w_3 &= \frac{n_3}{N} = (1-\theta)^2 \text{ (genotype frequency for wildtype homozygotes)},
\end{aligned}$$

where θ is the minor allele frequency (MAF).

We assume the mean gene expression levels for the 3 groups are

$$\begin{aligned}
\mu_1 &= a - \delta_1 \text{ (for mutation homozygotes)},\\
\mu_2 &= a \text{ (for heterozygotes)},\\
\mu_3 &= a + \delta_2 \text{ (for wildtype homozygote)}.
\end{aligned}$$

That is,

$$\begin{aligned}
\mu_2 - \mu_1 &= \delta_1,\\
\mu_3 - \mu_2 &= \delta_2.
\end{aligned}$$

Next, we would like to show that the non-centrality parameter λ depends only on δ_1 and δ_2, but not depends on a.

We can obtain

$$\begin{aligned}
\mu =& \omega_1\mu_1 + \omega_2\mu_2 + \omega_3\mu_3\\
=& \frac{n_1}{N}(a-\delta_1) + \frac{n_2}{N}a + \frac{n_3}{N}(a+\delta_2)\\
=& a\left(\frac{n_1}{N} + \frac{n_2}{N} + \frac{n_3}{N}\right) - \frac{n_1}{N}\delta_1 + \frac{n_3}{N}\delta_2\\
=& a - \frac{n_1}{N}\delta_1 + \frac{n_3}{N}\delta_2.
\end{aligned}$$

Hence,

$$\begin{aligned}\mu_1 - \mu =& (a - \delta_1) - \left(a - \frac{n_1}{N}\delta_1 + \frac{n_3}{N}\delta_2\right)\\ =& - \delta_1 + \frac{n_1}{N}\delta_1 - \frac{n_3}{N}\delta_2\\ \mu_2 - \mu =& a - \left(a - \frac{n_1}{N}\delta_1 + \frac{n_3}{N}\delta_2\right)\\ =& \frac{n_1}{N}\delta_1 - \frac{n_3}{N}\delta_2\\ \mu_3 - \mu =& (a + \delta_2) - \left(a - \frac{n_1}{N}\delta_1 + \frac{n_3}{N}\delta_2\right)\\ =& \delta_2 + \frac{n_1}{N}\delta_1 - \frac{n_3}{N}\delta_2.\end{aligned}$$

Hence, the non-centrality parameter λ depends only on δ_1 and δ_2, but not depends on a. Therefore, we can set $a = 0$ when we do programming. That is, we can set

$$\begin{aligned}&\mu_1 = -\delta_1 \text{ (for mutation homozygotes)},\\ &\mu_2 = 0 \text{ (for heterozygotes)},\\ &\mu_3 = \delta_2 \text{ (for wildtype homozygote)}.\end{aligned} \tag{10}$$

Next, we simplify the expression of λ.

Denote

$$p = \theta, q = 1 - p.$$

Then we have

$$w_1 = p^2, w_2 = 2pq, w_3 = q^2.$$

The overall mean can be rewritten as

$$\begin{aligned}\mu =& w_1\mu_1 + w_2\mu_2 + w_3\mu 3\\ =& p^2(-\delta_1) + q^2\delta_2\\ =& q^2\delta_2 - p^2\delta_1.\end{aligned} \tag{11}$$

Denote

$$\xi = \sum_{i=1}^{3} w_i \left(\mu_i - \mu\right)^2.$$

We can get

$$\begin{aligned}
\xi =& \sum_{i=1}^{3} w_i \left(\mu_i^2 - 2\mu_i\mu + \mu^2\right) \\
=& \sum_{i=1}^{3} \left[w_i\mu_i^2 - 2w_i\mu_i\mu + w_i\mu^2\right] \\
=& \sum_{i=1}^{3} w_i\mu_i^2 - 2\mu \sum_{i=1}^{3} w_i\mu_i + \mu^2 \sum_{i=1}^{3} w_i \\
=& \sum_{i=1}^{3} w_i\mu_i^2 - 2\mu^2 + \mu^2 \\
=& \sum_{i=1}^{3} w_i\mu_i^2 - \mu^2 \\
=& w_1\mu_1^2 + w_3\mu_3^2 - \mu^2 \\
=& p^2\delta_1^2 + q^2\delta_2^2 - \left(q^2\delta_2 - p^2\delta_1\right)^2 \\
=& p^2\delta_1^2 + q^2\delta_2^2 - \left(q^4\delta_2^2 + p^4\delta_1^2 - 2p^2q^2\delta_1\delta_2\right) \\
=& \left(p^2 - p^4\right)\delta_1^2 + \left(q^2 - q^4\right)\delta_2^2 + 2p^2q^2\delta_1\delta_2 \\
=& p^2(1-p)(1+p)\delta_1^2 + q^2(1-q)(1+q)\delta_2^2 + 2p^2q^2\delta_1\delta_2 \\
=& p^2q(1+p)\delta_1^2 + q^2p(1+q)\delta_2^2 + 2p^2q^2\delta_1\delta_2.
\end{aligned}$$

Hence,

$$\lambda = \frac{N}{\sigma^2}\left[p^2q(1+p)\delta_1^2 + q^2p(1+q)\delta_2^2 + 2p^2q^2\delta_1\delta_2\right]. \tag{12}$$

2 Power Calculation for Bulk Tissue eQTL Based on SLR

2.1 Introduction

If we would like to test linear relationship between genotype of a SNP and expression of a gene, we can use simple linear regression:

$$\begin{aligned}
y_i =& \beta_0 + \beta_1 x_i + \epsilon_i,\ \epsilon_i \sim \mathrm{N}\left(0, \sigma^2\right), \\
i =& 1, \ldots, n,
\end{aligned} \tag{13}$$

where y_i is the expression level of the gene for the subject i and x_i is the genotype of the i-th subject by using additive coding. That is, $x_i = 0$ for wild-type homozygote (containing zero minor allele), $x_i = 1$ for heterozygote (containing one minor allele), and $x_i = 2$ for mutation homozygote (containing two minor allele).

2.2 Mean and variance of additive coded genotype

Denote A as major allele and a as minor allele. Denote X as the random variable recoding the additive coded genotype and denote the genotype frequencies as

$$\begin{aligned} p_0 =& Pr(AA) = Pr(X = 0), \\ p_1 =& Pr(Aa) = Pr(X = 1), \\ p_2 =& Pr(aa) = Pr(X = 2). \end{aligned} \tag{14}$$

Then we can calculate the mean and variance of the genotype

$$\begin{aligned} \mu_x =& E(X) \\ =& 0 \cdot Pr(X = 0) + 1 \cdot Pr(X = 1) + 2 \cdot Pr(X = 2) \\ =& p_1 + 2p_2, \\ \sigma_x^2 =& E(X^2) - [E(X)]^2 \\ =& \left[0^2 \cdot Pr(X = 0) + 1^2 \cdot Pr(X = 1) + 2^2 \cdot Pr(X = 2)\right] - \mu_x^2 \\ =& p_1 + 4p_2 - \mu_x^2. \end{aligned} \tag{15}$$

Equivalently,

$$\begin{aligned} p_1 =& 2\mu_x - \mu_x^2 - \sigma_x^2, \\ p_2 =& \frac{(\sigma_x^2 - \mu_x + \mu_x^2)}{2}. \end{aligned} \tag{16}$$

2.2.1 Hardy-Weinberg equilibrium

Denote the minor allele frequency (MAF) as $\theta = Pr(a)$. Under Hardy-Weinberg equilibrium, we have the following results:

$$\begin{aligned} p_0 =& Pr(AA) = (1 - \theta)^2, \\ p_1 =& Pr(Aa) = 2\theta(1 - \theta), \\ p_2 =& Pr(aa) = \theta^2. \end{aligned} \tag{17}$$

In this case, we have

$$\begin{aligned}\mu_x =& 2\theta,\\ \sigma_x^2 =& 2(1-\theta)\theta,\end{aligned} \tag{18}$$

and

$$\sigma_x^2 = \mu_x - \mu_x^2/2. \tag{19}$$

2.3 Power Calculation of eQTL based on simple linear regression

2.3.1 Power calculation for simple linear regression

The exact power calculation formula derived in this section is an improvement of the appro-lambdamate power calculation formula derived in Dupont and Plummer (1998). For simple linear regression (13), the estimate $\hat{\beta}_1$ of slope is

$$\hat{\beta}_1 = \frac{\sum_{i=1}^n (x_i - \bar{x})(y_i - \bar{y})}{\sum_{i=1}^n (x_i - \bar{x})^2}, \tag{20}$$

where

$$\bar{x} = \frac{1}{n}\sum_{i=1}^n x_i,\ \bar{y} = \frac{1}{n}\sum_{i=1}^n y_i.$$

Denote

$$\begin{aligned}L_{xx} =& \sum_{i=1}^n (x_i - \bar{x})^2 = \sum_{i=1}^n x_i^2 - n(\bar{x})^2\\ L_{xy} =& \sum_{i=1}^n (x_i - \bar{x})(y_i - \bar{y}) = \sum_{i=1}^n x_i y_i - n\bar{x}\bar{y}.\end{aligned}$$

Then

$$\hat{\beta}_1 = \frac{L_{xy}}{L_{xx}}.$$

We are interested in testing the hypotheses:

$$H_0 : \beta_1 = 0 \text{ versus } H_1 : \beta_1 = \delta(\delta \neq 0). \tag{21}$$

Under the alternative hypothesis H_1,

$$\hat{\beta}_1 \sim \mathrm{N}\left(\delta,\ \frac{\sigma^2}{L_{xx}}\right). \tag{22}$$

We can construct the following test statistic to test if $\beta_1 = 0$:

$$t = \frac{\hat{\beta}_1}{\sqrt{\hat{\sigma}^2 / L_{xx}}},$$

where

$$\hat{\sigma}^2 = \frac{1}{n-2} \sum_{i=1}^{n} (y_i - \hat{y}_i)^2$$

is the unbiased estimate of σ^2 (i.e., $E\left(\hat{\sigma}^2\right) = \sigma^2$), and

$$\hat{y}_i = \hat{\beta}_0 + \hat{\beta}_1 x_i$$

and

$$\hat{\beta}_0 = \bar{y} - \hat{\beta}_1 \bar{x}.$$

It can be shown that

$$(n-2)\frac{\hat{\sigma}^2}{\sigma^2} \sim \chi^2_{n-2}. \tag{23}$$

Under $H_0 : \beta_1 = 0$,

$$\frac{\hat{\beta}_1}{\sqrt{\sigma^2 / L_{xx}}} \sim N(0, 1).$$

It can be shown that $\hat{\beta}_1$ is independent of $\hat{\sigma}^2$.

Note that if $Z \sim N(0, 1)$, $V \sim \chi^2_\nu$, and Z and V independent, then $Z/\sqrt{V/\nu} \sim t_\nu$, where t_ν is the t distribution with ν degrees of freedom. Hence, we have

$$t = \frac{\hat{\beta}_1}{\sqrt{\hat{\sigma}^2 / L_{xx}}} = \frac{\frac{\hat{\beta}_1}{\sqrt{\sigma^2 / L_{xx}}}}{\sqrt{(n-2)\frac{\hat{\sigma}^2}{\sigma^2}/(n-2)}} \overset{H_0}{\sim} t_{n-2}.$$

Hence, the Type I error rate α for two-sided test is

$$\alpha = Pr\left(|t| > t_{n-2}(\alpha/2) | H_0\right), \tag{24}$$

where $t_{n-2}(\alpha/2)$ is the upper $100(\alpha/2)\%$ percentile of the t distribution with degree of freedom $n-2$.

We use the following fact: if $Z \sim N(0,1)$, $V \sim \chi^2_\nu$, and Z and V are independent, then

$$\frac{Z+\lambda}{\sqrt{V/\nu}} \sim t_{\nu,\lambda},$$

where $t_{\nu,\lambda}$ is the non-central t distribution with ν degrees of freedom and non-centrality parameter λ.

Hence, based on (22), we have

$$\frac{\hat{\beta}_1}{\sqrt{\sigma^2/L_{xx}}} \overset{H_1}{\sim} N(\lambda, 1),$$

where

$$\lambda = \frac{\delta}{\sqrt{\sigma^2/L_{xx}}} = \frac{\delta}{\sqrt{\sigma^2/\left[(n-1)\tilde{\sigma}_x^2\right]}}, \tag{25}$$

and $\tilde{\sigma}_x^2$ is an unbiased estimate of σ_x^2:

$$\tilde{\sigma}_x^2 = \frac{1}{n-1}\sum_{i=1}^{n}(x_i - \bar{x})^2.$$

Under H_1 we have

$$t = \frac{\hat{\beta}_1}{\sqrt{\hat{\sigma}^2/L_{xx}}} = \frac{\frac{\hat{\beta}_1}{\sqrt{\sigma^2/L_{xx}}}}{\sqrt{(n-2)\frac{\hat{\sigma}^2}{\sigma^2}/(n-2)}} \overset{H_1}{\sim} t_{n-2,\lambda}. \tag{26}$$

Hence, the exact power is calculated as

$$\begin{aligned} 1-\beta =& Pr\left(|t| > t_{n-2}(\alpha/2)|H_1\right) \\ =& Pr\left(t > t_{n-2}(\alpha/2)|H_1\right) + Pr\left(t < -t_{n-2}(\alpha/2)|H_1\right) \\ =& 1 - T_{n-2,\lambda}\left[t_{n-2}(\alpha/2)\right] + T_{n-2,\lambda}\left[-t_{n-2}(\alpha/2)\right], \end{aligned} \tag{27}$$

where $T_{n-2,\lambda}(a)$ is the value at a of CDF of non-central t distribution with $(n-2)$ degrees of freedom and non-centrality parameter λ.

Formula (25) shows that λ depends on the standard deviation σ of random error, which is not easy to estimate or to set its value in design stage of a study. Instead, the standard deviation of the outcome σ_y is relative easier to estimate or set based on historical data.

Formulas (1) and (2) in Dupont and Plummer (1998)[1] describe the relationships among

the slope β_1, the variance of the outcome σ_y^2, the variance of predictor σ_x^2, and the variance of the random error σ^2:

$$\sigma^2 = \sigma_y^2 - \beta_1^2 \sigma_x^2. \tag{28}$$

Hence, we can rewrite the non-centrality parameter λ as

$$\lambda = \frac{\delta}{\sqrt{\left(\sigma_y^2 - \delta^2 2(1-\hat{\theta})\hat{\theta}\right) / \left[(n-1)2(1-\hat{\theta})\hat{\theta}\right]}}. \tag{29}$$

Since $\sigma^2 > 0$, we require

$$\sigma_y^2 - \delta^2 2(1-\theta)\theta > 0.$$

We can get

$$-\frac{\sigma_y}{\sqrt{2\theta(1-\theta)}} < \delta < \frac{\sigma_y}{\sqrt{2\theta(1-\theta)}}. \tag{30}$$

and

$$\left(\theta - \frac{1}{2}\right)^2 > \frac{1}{4} - \frac{\sigma_y^2}{2\delta^2}.$$

If $\frac{1}{4} - \frac{\sigma_y^2}{2\delta^2} > 0$, we then require

$$\left(\theta - \frac{1}{2}\right) > \sqrt{\frac{1}{4} - \frac{\sigma_y^2}{2\delta^2}}$$

or

$$\left(\theta - \frac{1}{2}\right) < -\sqrt{\frac{1}{4} - \frac{\sigma_y^2}{2\delta^2}}$$

Since $\theta < 0.5$, we require

$$0 < \theta < \frac{1}{2} - \sqrt{\frac{1}{4} - \frac{\sigma_y^2}{2\delta^2}}. \tag{31}$$

3 Power Calculation for Single-Cell eQTL

3.1 A Linear Mixed Effects Model

We are interested in testing if a SNP is associated with the expression of a gene based on single cell RNAseq data, which contain n subjects. For each subject, we obtain m cells. For each cell, we measured the expression of G genes.

We assume the following linear mixed effects model to characterize the association between genotype of a given SNP and expression of a given gene:

$$\begin{aligned} y_{ij} =& \beta_{0i} + \beta_1 x_i + \epsilon_{ij}, \\ \beta_{0i} \sim& N\left(\beta_0, \sigma_\beta^2\right), \\ \epsilon_{ij} \sim& N\left(0, \sigma^2\right), \\ i =& 1, \ldots, n, \\ j =& 1, \ldots, m, \end{aligned} \tag{32}$$

where the random intercepts β_{0i} and the random error terms ϵ_{ij} are independent, n is the number of subjects, m is the number of cells per subject, y_{ij} is the gene expression of the j-th cell for the i-th subject, and x_i is the genotype for the i-th subject. $x_i = 0$ indicates that the i-th subject is a wildtype homozygote, $x_i = 1$ indicates that the i-th subject is a heterozygote, and $x_i = 2$ indicates that the i-th subject is a mutation homozygote.

Note that the random intercept β_{0i} helps incorporate intra-class correlation between y_{ij} and y_{ik}, for $j \neq k$. The covariance between y_{ij} and y_{ik} is

$$\begin{aligned} Cov\left(y_{ij}, y_{ik}\right) =& Cov\left(\beta_{0i} + \beta_1 x_i + \epsilon_{ij}, \beta_{0i} + \beta_1 x_i + \epsilon_{ik}\right) \\ =& Var\left(\beta_{0i}\right) \\ =& \sigma_\beta^2. \end{aligned}$$

3.2 Hypotheses

The mean gene expression for the 3 genotypes are

$$\begin{aligned} E\left(y_{ij}\right) =& \beta_0 && \text{if subject } i \text{ is a wildtype homozygote,} \\ E\left(y_{ij}\right) =& \beta_0 + \beta_1 && \text{if subject } i \text{ is a heterozygote,} \\ E\left(y_{ij}\right) =& \beta_0 + 2\beta_1 && \text{if subject } i \text{ is a mutation homozygote.} \end{aligned}$$

If the slope $\beta_1 = 0$, then all three genotypes have the same mean gene expression $E\left(y_{ij}\right) = \beta_0$. Hence, to test if a SNP is associated with a gene is equivalent to test if the slope $\beta_1 = 0$ or not.

We would like to test the following null hypothesis (H_0) and alternative hypothesis (H_1):

$$
\begin{aligned}
H_0 :& \beta_1 = 0, \\
H_1 :& \beta_1 = \delta,
\end{aligned}
\tag{33}
$$

where $\delta \neq 0$.

3.3 Power calculation formula

For a given SNP-gene pair, we derived the power calculation formula for testing Hypotheses (33) as shown below:

$$
power = 1 - \Phi\left(z_{\alpha/2} - \frac{\hat{\sigma}_x}{\sigma_y}\frac{\delta\sqrt{m(n-1)}}{\sqrt{1+(m-1)\rho}}\right) + \Phi\left(-z_{\alpha/2} - \frac{\hat{\sigma}_x}{\sigma_y}\frac{\delta\sqrt{m(n-1)}}{\sqrt{1+(m-1)\rho}}\right), \tag{34}
$$

where α is the type I error rate, $z_{\alpha/2}$ is the upper $100\alpha/2$ percentile of the standard normal distribution $N(0,1)$, $\sigma_y = \sqrt{\sigma_\beta^2 + \sigma^2}$ is the standard deviation of y_{ij} (i.e., $\sigma_y = \sqrt{Var\left(y_{ij}\right)}$), $\hat{\sigma}_x = \sqrt{\sum_{i=1}^n \left(x_i - \bar{x}\right)^2 / (n-1)}$ is the sample standard deviation of the predictor (i.e., genotype) x_i, and $\rho = \sigma_\beta^2 / \left(\sigma_\beta^2 + \sigma^2\right)$ is the intra-class correlation (i.e., correlation between y_{ij} and y_{ik}).

The power calculation formula for testing Hypotheses (33) for genotypes under Hardy-Weinberg Equilibrium is:

$$
\begin{aligned}
power =& 1 - \Phi\left(z_{\alpha/2} - \frac{\sqrt{2\theta\left(1-\theta\right)}}{\sigma_y}\frac{\delta\sqrt{m(n-1)}}{\sqrt{1+(m-1)\rho}}\right) \\
&+ \Phi\left(-z_{\alpha/2} - \frac{\sqrt{2\theta\left(1-\theta\right)}}{\sigma_y}\frac{\delta\sqrt{m(n-1)}}{\sqrt{1+(m-1)\rho}}\right).
\end{aligned}
\tag{35}
$$

where θ is the minor allele frequency of the SNP.

The details of the derivations are shown in Appendices.

3.4 Discussion

Note that we assume the gene expression levels y_{ij} are normally distributed. However, RNAseq data are counts and many counts are zero. So y_{ij} could not be normally distributed. We might use data transformations (e.g., R package *countTransformers*[5] devel-

oped in Zhang et al., 2019[6]). However, usually we can only make sure the median and mean are close, while the transformed data are still not normally distributed. The effect of using normal assumption to fit non-normal data is false-positive inflation. So the power calculated will be higher than the true power. In future, we will derive power calculation formula based on mixed effects negative binomial regressions that are popular in fitting RNAseq data. The challenge is that no closed-form power calculation formulas can be derived for negative binomial regressions, let alone mixed effects negative binomial regressions. We will try to derive approximate power-calculation formulas and to use simulation approach.

4 Power Calculation for Single-Cell eQTL via Simulation based on ZINB Mixed Effects Regression Model

One approach to analyze single-cell RNAseq (scRNAseq) data is to directly use the read counts as outcome variable in generalized linear regression. For example, Zheng (2016)[7] used zero-inflated negative binomial (ZINB) regression model to detect differentially expressed genes based on scRNAseq and to simulate scRNAseq data. Vieth et al. (2017)[4] also mentioned to use ZINB for differential expression (DE) analysis based on scRNAseq data.

As the best of our knowledge, no studies have used ZINB mixed effects regression model yet to calculate power of single cell expression quantitative loci (sc-eQTL) analysis. Since there is no closed-form power-calculation formula for ZINB mixed effects regression model, we use a simulation approach.

4.1 ZINB mixed effects regression model

We assume that the gene expression levels (read counts) follow zero-inflated negative binomial distribution. Denote Y_{ij} as the read counts for the j-th cell of the i-th subject, $i = 1, \ldots, n$, $j = 1, \ldots, m$, n is the number of subjects, and m is the number of cells per subject. Denote p as the probability that $Y_{ij} = 0$ is an excess zero. With probability $1 - p$, Y_{ij} follows a negative binomial distribution $NB(\mu, \theta)$, where μ is the mean (i.e., $\mu = E(Y_{ij})$) and θ is the dispersion parameter. The variance of the NB distribution is $\mu + \mu^2/\theta$. The relationship between gene expression and genotype for the i-th subject is characterized by the equation

$$\mu_i = \exp(\beta_{0i} + \beta_1 x_i), \tag{36}$$

where β_{0i} is the random intercept following a normal distribution $N(\beta_0, \sigma^2)$ to account for within-subject correlation of gene expression, β_0 is the mean of the random intercept, σ is the standard deviation of the random intercept, β_1 is the slope, and x_i is the additive-coded genotype for the SNP with minor allele frequency MAF.

We assume that the SNP satisfies the Hardy-Weinberg Equilibrium. That is, the probabilities of the 3 genotypes $(0, 1, 2)$ are $(1-MAF)^2$, $2MAF(1-MAF)$, MAF^2, respectively.

For simplicity, we assume that excess zeros are caused by technical issues, hence are not related to genotypes.

4.2 Power calculation via simulation

To calculate power via simulation, we perform the following steps:

Step 1 Simulate $nSim$ datasets. Each dataset contains read counts of one gene and genotype of one SNP for n subjects, each with m cells.

Step 2 Simulate genotypes for the SNP. We assume that each subject's genotypes are the same across cells. We also assume the Hardy-Weinberg Equilibrium.

Step 3 The read counts of the gene follows a mixture of 2-component distributions. One component takes only one value: zero. The other component is negative binomial distribution, which takes non-negative values 0, 1, 2, ... The log mean of the negative binomial distribution is linear function of the genotype (Formula 36).

Step 4 For each simulated dataset, we fit data via ZINB mixed effects regression model. The p-value for testing if the slope β_1 for genotype is equal to zero will be calculated.

Step 5 The proportion of p-values $< \alpha$ is the estimated power, where $\alpha = 0.05/nTests$, and $nTests$ is the number of all (SNP, gene) pairs in sc-eQTL analysis.

4.3 Parameter estimates for ZINB mixed effects regression model

Currently, there are two R packages (*GLMMadaptive* and *glmmTMB*) in CRAN that can fit ZINB mixed effects. We included both R packages in powerEQTL. By default, *GLMMadaptive* is used to obtain the p-value for testing if the slope β_1 is equal to zero or not.

4.4 Parallel computing

The simulation approach is computational intensive. Parallel computing would improve the speed. We used the function *mclapply* in the R packagle *parallel* to calculate power via parallel computing.

Appendix

A Vector representation

In this section, we derive the vector representations of Model (32).

We represent β_{0i} by

$$\beta_{0i} = \beta_0 + \xi_i,$$

where

$$\xi_i \sim N\left(0, \sigma_\beta^2\right).$$

Denote

$$e_{ij} = \xi_i + \epsilon_{ij}.$$

Then

$$e_{ij} \sim N\left(0, \sigma_\beta^2 + \sigma^2\right).$$

Denote

$$\sigma_y^2 = \sigma_\beta^2 + \sigma^2.$$

Note that

$$Var\left(y_{ij}\right) = Var\left(\beta_{0i}\right) + Var\left(\epsilon_{ij}\right) = \sigma_\beta^2 + \sigma^2 = \sigma_y^2. \tag{A1}$$

Model (32) can be rewritten as

$$\begin{aligned} y_{ij} =& \beta_0 + \beta_1 x_i + e_{ij}, \\ e_{ij} \sim& N\left(0, \sigma_y^2\right), \\ i =& 1, \ldots, n, \\ j =& 1, \ldots, m, \end{aligned} \tag{A2}$$

with $Cov\left(y_{ij}, y_{ik}\right) = \sigma_\beta^2$ for $j \neq k$.

Denote

$$\boldsymbol{y}_i = \begin{pmatrix} y_{i1} \\ \vdots \\ y_{im} \end{pmatrix}, \quad \boldsymbol{e}_i = \begin{pmatrix} e_{i1} \\ \vdots \\ e_{im} \end{pmatrix}, \quad \boldsymbol{u}_i = \begin{pmatrix} 1 \\ x_i \end{pmatrix}, \quad \boldsymbol{\beta} = \begin{pmatrix} \beta_0 \\ \beta_1 \end{pmatrix}.$$

Also, denote $\mathbf{1}_m$ as the $m \times 1$ vector of ones, $\mathbf{0}_m$ as the $m \times 1$ vector of zeros, and $\boldsymbol{I}_m$ as the

$m \times m$ identity matrix.

Model (A2) can be rewritten as

$$\begin{aligned} \boldsymbol{y}_i =& \mathbf{1}_m \boldsymbol{u}_i^T \boldsymbol{\beta} + \boldsymbol{e}_i, \\ \boldsymbol{e}_i \sim& N\left(\mathbf{0}_m, \boldsymbol{\Sigma}\right), \\ i =& 1, \ldots, n, \end{aligned} \tag{A3}$$

where $\boldsymbol{y}_i$ is a $m \times 1$ vector, $\mathbf{1}_m$ is a $m \times 1$ vector, $\boldsymbol{u}_i^T$ is a 1×2 vector, $\boldsymbol{\beta}$ is a 2×1 vector, $\boldsymbol{e}_i$ is a $m \times 1$ vector, $\boldsymbol{\Sigma}$ is a $m \times m$ matrix

$$\boldsymbol{\Sigma} = \begin{pmatrix} \sigma_y^2 & & \sigma_\beta^2 \\ & \ddots & \\ \sigma_\beta^2 & & \sigma_y^2 \end{pmatrix} = \sigma_y^2 \begin{pmatrix} 1 & & \rho \\ & \ddots & \\ \rho & & 1 \end{pmatrix},$$

and

$$\rho = \frac{\sigma_\beta^2}{\sigma_y^2} = \frac{\sigma_\beta^2}{\sigma_\beta^2 + \sigma^2}.$$

Note that ρ is also called intra-class correlation (ICC).

Denote

$$\boldsymbol{R} = \begin{pmatrix} 1 & & \rho \\ & \ddots & \\ \rho & & 1 \end{pmatrix}.$$

Then

$$\boldsymbol{\Sigma} = \sigma_y^2 \boldsymbol{R}.$$

Denote

$$\boldsymbol{y} = \begin{pmatrix} \boldsymbol{y}_1 \\ \vdots \\ \boldsymbol{y}_n \end{pmatrix}, \quad \boldsymbol{U} = \begin{pmatrix} \mathbf{1}_m \boldsymbol{u}_1^T \\ \vdots \\ \mathbf{1}_m \boldsymbol{u}_n^T \end{pmatrix}, \quad \boldsymbol{e} = \begin{pmatrix} \boldsymbol{e}_1 \\ \vdots \\ \boldsymbol{e}_n \end{pmatrix}, \quad \boldsymbol{\Omega} = \begin{pmatrix} \boldsymbol{\Sigma} & & \mathbf{0} \\ & \ddots & \\ \mathbf{0} & & \boldsymbol{\Sigma} \end{pmatrix}.$$

Model (A3) can be rewritten as

$$\begin{aligned} \boldsymbol{y} =& \boldsymbol{U}\boldsymbol{\beta} + \boldsymbol{e}, \\ \boldsymbol{e} \sim& N\left(\mathbf{0}_{(n \times m) \times 1}, \boldsymbol{\Omega}\right), \end{aligned} \tag{A4}$$

where $\boldsymbol{y}$ is a $(n \times m) \times 1$ vector, $\boldsymbol{U}$ is a $(n \times m) \times 2$ matrix, $\boldsymbol{\beta}$ is a 2×1 vector, and $\boldsymbol{e}$ is a $(n \times m) \times 1$ vector.

B Generalized least squares estimate when variance-covariance matrix is known

Denote the weighted distance between $\boldsymbol{y}$ and the linear part $\boldsymbol{U\beta}$ in Model (A4) as

$$\begin{aligned} g(\boldsymbol{\beta}) &= (\boldsymbol{y} - \boldsymbol{U\beta})^T \boldsymbol{\Omega}^{-1} (\boldsymbol{y} - \boldsymbol{U\beta}) \\ &= \boldsymbol{y}^T \boldsymbol{\Omega}^{-1} \boldsymbol{y} - 2\boldsymbol{y}^T \boldsymbol{\Omega}^{-1} \boldsymbol{U\beta} + \boldsymbol{\beta}^T \boldsymbol{U}^T \boldsymbol{\Omega}^{-1} \boldsymbol{U\beta}. \end{aligned} \tag{A5}$$

The generalized least squares estimate of $\boldsymbol{\beta}$ when the variance-covariance matrix $\boldsymbol{\Omega}$ is known is obtained by solving the following minimization problem:

$$min_{\boldsymbol{\beta}} g(\boldsymbol{\beta}). \tag{A6}$$

The first order partial derivative of $g(\boldsymbol{\beta})$ to $\boldsymbol{\beta}$ is

$$\frac{\partial g(\boldsymbol{\beta})}{\partial \boldsymbol{\beta}} = -2\boldsymbol{U}^T \boldsymbol{\Omega}^{-1} \boldsymbol{y} + 2\boldsymbol{U}^T \boldsymbol{\Omega}^{-1} \boldsymbol{U\beta}.$$

Let the first partial derivative be equal to zero, we can get

$$\hat{\boldsymbol{\beta}} = \left(\boldsymbol{U}^T \boldsymbol{\Omega}^{-1} \boldsymbol{U}\right)^{-1} \boldsymbol{U}^T \boldsymbol{\Omega}^{-1} \boldsymbol{y}. \tag{A7}$$

The second order partial derivative of $g(\boldsymbol{\beta})$ to $\boldsymbol{\beta}$ is

$$\frac{\partial^2 g(\boldsymbol{\beta})}{\partial \boldsymbol{\beta} \partial \boldsymbol{\beta}^T} = 2\boldsymbol{U}^T \boldsymbol{\Omega}^{-1} \boldsymbol{U},$$

which is positive definite. Hence, $\hat{\boldsymbol{\beta}}$ minimizes, not maximize, the objective function $g(\boldsymbol{\beta})$. We call $\hat{\boldsymbol{\beta}}$ the generalized least squares estimate of $\boldsymbol{\beta}$ when $\boldsymbol{\Omega}$ is known.

C Mean, variance, and distribution of the generalized least squares estimate

The mean of $\hat{\boldsymbol{\beta}}$ is

$$E\left(\hat{\boldsymbol{\beta}}\right) = \left(\boldsymbol{U}^T\boldsymbol{\Omega}^{-1}\boldsymbol{U}\right)^{-1}\boldsymbol{U}^T\boldsymbol{\Omega}^{-1}E\left(\boldsymbol{y}\right)$$
$$= \left(\boldsymbol{U}^T\boldsymbol{\Omega}^{-1}\boldsymbol{U}\right)^{-1}\boldsymbol{U}^T\boldsymbol{\Omega}^{-1}\boldsymbol{U}\boldsymbol{\beta}$$
$$=\boldsymbol{\beta}.$$

That is $\hat{\boldsymbol{\beta}}$ is an unbiased estimate of $\boldsymbol{\beta}$.

The variance-covariance matrix of $\hat{\boldsymbol{\beta}}$ is

$$\begin{aligned} Cov\left(\hat{\boldsymbol{\beta}}\right) &= \left(\boldsymbol{U}^T\boldsymbol{\Omega}^{-1}\boldsymbol{U}\right)^{-1}\boldsymbol{U}^T\boldsymbol{\Omega}^{-1}Cov\left(\boldsymbol{y}\right)\boldsymbol{\Omega}^{-1}\boldsymbol{U}\left(\boldsymbol{U}^T\boldsymbol{\Omega}^{-1}\boldsymbol{U}\right)^{-1} \\ &= \left(\boldsymbol{U}^T\boldsymbol{\Omega}^{-1}\boldsymbol{U}\right)^{-1}\boldsymbol{U}^T\boldsymbol{\Omega}^{-1}\boldsymbol{\Omega}\boldsymbol{\Omega}^{-1}\boldsymbol{U}\left(\boldsymbol{U}^T\boldsymbol{\Omega}^{-1}\boldsymbol{U}\right)^{-1} \\ &= \left(\boldsymbol{U}^T\boldsymbol{\Omega}^{-1}\boldsymbol{U}\right)^{-1}. \end{aligned} \tag{A8}$$

Since $\boldsymbol{y}$ is normally distributed, hence $\hat{\boldsymbol{\beta}}$ is also normally distributed.

D Calculating the power for testing if the slope is equal to zero

To test the null hypothesis $H_0 : \beta_1 = 0$ versus the alternative hypothesis $H_1 : \beta_1 = \delta$, where $\delta \neq 0$, we can construct the test statistic

$$Z = \frac{\hat{\beta}_1}{\sqrt{Var\left(\hat{\beta}_1\right)}}. \tag{A9}$$

Note that we assume $\boldsymbol{\Omega}$ is known. Hence, Z is a statistic. Otherwise (i.e., if $\boldsymbol{\Omega}$ is unknown), Z is not a statistic since it contains unknown parameters.

Under $H_0 : \beta_1 = 0$, Z follows standard normal distribution $N(0, 1)$. Under $H_1 : \beta_1 = \delta$,

where $\delta \neq 0$,

$$\frac{\hat{\beta}_1 - \delta}{\sqrt{Var\left(\hat{\beta}_1\right)}} \sim N(0,1).$$

If we set the decision rule as

$$\text{reject } H_0 : \beta_1 = 0 \text{ if } |Z| > z_{\alpha/2},$$

then the Type I error rate is equal to α, where $z_{\alpha/2}$ is the upper $100\alpha/2$ percentile. That is,

$$Pr\left(|Z| > z_{\alpha/2}|H_0\right) = \alpha$$

We also can calculate the power for testing he null hypothesis $H_0 : \beta_1 = 0$ versus the alternative hypothesis $H_1 : \beta_1 = \delta$, where $\delta \neq 0$.

$$\begin{aligned} power =& Pr\left(|Z| > z_{\alpha/2}|H_1\right) \\ =& Pr\left(Z > z_{\alpha/2}|H_1\right) + Pr\left(Z < -z_{\alpha/2}|H_1\right). \end{aligned}$$

We can get

$$\begin{aligned} & Pr\left(Z > z_{\alpha/2}|H_1\right) \\ =& Pr\left(\left.\frac{\hat{\beta}_1}{\sqrt{Var\left(\hat{\beta}_1\right)}} > z_{\alpha/2}\right| H_1\right) \\ =& Pr\left(\left.\frac{\hat{\beta}_1 - \delta + \delta}{\sqrt{Var\left(\hat{\beta}_1\right)}} > z_{\alpha/2}\right| H_1\right) \\ =& Pr\left(\left.\frac{\hat{\beta}_1 - \delta}{\sqrt{Var\left(\hat{\beta}_1\right)}} > z_{\alpha/2} - \frac{\delta}{\sqrt{Var\left(\hat{\beta}_1\right)}}\right| H_1\right) \\ =& 1 - \Phi\left(z_{\alpha/2} - \frac{\delta}{\sqrt{Var\left(\hat{\beta}_1\right)}}\right), \end{aligned}$$

where Φ is the cumulative distribution function of the standard normal distribution $N(0,1)$.

Similarly, we can get

$$\begin{aligned}
&Pr\left(Z < -z_{\alpha/2}|H_1\right)\\
=&Pr\left(\left.\frac{\hat{\beta}_1}{\sqrt{Var\left(\hat{\beta}_1\right)}} < -z_{\alpha/2}\right| H_1\right)\\
=&Pr\left(\left.\frac{\hat{\beta}_1-\delta+\delta}{\sqrt{Var\left(\hat{\beta}_1\right)}} < -z_{\alpha/2}\right| H_1\right)\\
=&Pr\left(\left.\frac{\hat{\beta}_1-\delta}{\sqrt{Var\left(\hat{\beta}_1\right)}} < -z_{\alpha/2}-\frac{\delta}{\sqrt{Var\left(\hat{\beta}_1\right)}}\right| H_1\right)\\
=&\Phi\left(-z_{\alpha/2}-\frac{\delta}{\sqrt{Var\left(\hat{\beta}_1\right)}}\right).
\end{aligned}$$

Therefore the power is

$$power = 1-\Phi\left(z_{\alpha/2}-\frac{\delta}{\sqrt{Var\left(\hat{\beta}_1\right)}}\right)+\Phi\left(-z_{\alpha/2}-\frac{\delta}{\sqrt{Var\left(\hat{\beta}_1\right)}}\right). \qquad \text{(A10)}$$

E Calculation of the variance of the slope estimate

Based on Formula (A8), we have

$$Cov\left(\hat{\boldsymbol{\beta}}\right) = \left(\boldsymbol{U}^T\boldsymbol{\Omega}^{-1}\boldsymbol{U}\right)^{-1}.$$

We first calculate $\boldsymbol{\Omega}^{-1}$. We have

$$\boldsymbol{\Omega}^{-1} = \begin{pmatrix} \boldsymbol{\Sigma}^{-1} & & \mathbf{0} \\ & \ddots & \\ \mathbf{0} & & \boldsymbol{\Sigma}^{-1} \end{pmatrix}.$$

We then calculate $\boldsymbol{\Sigma}^{-1}$. We have

$$\boldsymbol{\Sigma}^{-1} = \frac{1}{\sigma_y^2}\boldsymbol{R}^{-1}.$$

We next calculate $\boldsymbol{R}^{-1}$. We can rewrite $\boldsymbol{R}$ to

$$\boldsymbol{R} = (1-\rho)\left(\boldsymbol{I}_m + \frac{\rho}{1-\rho}\mathbf{1}_m\mathbf{1}_m^T\right).$$

Then we have

$$\boldsymbol{R}^{-1} = \frac{1}{(1-\rho)}\left(\boldsymbol{I}_m + \frac{\rho}{1-\rho}\mathbf{1}_m\mathbf{1}_m^T\right)^{-1}.$$

Based on the matrix theories, we have the following results

$$\left(\boldsymbol{A} + \boldsymbol{b}\boldsymbol{c}^T\right)^{-1} = \boldsymbol{A}^{-1} - \frac{\boldsymbol{A}^{-1}\boldsymbol{b}\boldsymbol{c}\boldsymbol{A}^{-1}}{1 + \boldsymbol{c}^T\boldsymbol{A}^{-1}\boldsymbol{b}},$$

where $\boldsymbol{A}$ is a matrix, $\boldsymbol{b}$ and $\boldsymbol{c}$ are vectors.

Let $\boldsymbol{A} = \boldsymbol{I}_m$, $\boldsymbol{b} = \frac{\rho}{1-\rho}\mathbf{1}_m$, $\boldsymbol{c} = \mathbf{1}_m$. We have $\boldsymbol{A}^{-1} = \boldsymbol{I}_m$, $\mathbf{1}_m^T\mathbf{1}_m = m$, and

$$\begin{aligned} &\left(\boldsymbol{I}_m + \frac{\rho}{1-\rho}\mathbf{1}_m\mathbf{1}_m^T\right)^{-1} \\ =&\boldsymbol{I}_m - \frac{\frac{\rho}{1-\rho}\mathbf{1}_m\mathbf{1}_m^T}{1 + \frac{\rho}{1-\rho}m} \\ =&\boldsymbol{I}_m - \frac{\rho\mathbf{1}_m\mathbf{1}_m^T}{(1-\rho) + \rho m} \\ =&\boldsymbol{I}_m - \frac{\rho\mathbf{1}_m\mathbf{1}_m^T}{1 + (m-1)\rho}. \end{aligned}$$

Hence, we have

$$\boldsymbol{R}^{-1} = \frac{1}{(1-\rho)}\left[\boldsymbol{I}_m - \frac{\rho\mathbf{1}_m\mathbf{1}_m^T}{1 + (m-1)\rho}\right]$$

and

$$\mathbf{\Sigma}^{-1} = \frac{1}{\sigma_y^2(1-\rho)}\left[\boldsymbol{I}_m - \frac{\rho\mathbf{1}_m\mathbf{1}_m^T}{1+(m-1)\rho}\right]. \tag{A11}$$

Next, we calculate

$$\begin{aligned}
\boldsymbol{U}^T\mathbf{\Omega}^{-1}\boldsymbol{U} &= \left(\boldsymbol{u}_1\mathbf{1}_m^T, \ldots, \boldsymbol{u}_n\mathbf{1}_m^T\right)\begin{pmatrix} \mathbf{\Sigma}^{-1} & & \mathbf{0} \\ & \ddots & \\ \mathbf{0} & & \mathbf{\Sigma}^{-1}\end{pmatrix}\begin{pmatrix} \mathbf{1}_m\boldsymbol{u}_1^T \vdots \\ \mathbf{1}_m\boldsymbol{u}_n^T \end{pmatrix} \\
&= \sum_{i=1}^n \boldsymbol{u}_i\mathbf{1}_m^T\mathbf{\Sigma}^{-1}\mathbf{1}_m\boldsymbol{u}_i^T \\
&= \mathbf{1}_m^T\mathbf{\Sigma}^{-1}\mathbf{1}_m\sum_{i=1}^n \boldsymbol{u}_i\boldsymbol{u}_i^T.
\end{aligned}$$

We have

$$\begin{aligned}
\mathbf{1}_m^T\mathbf{\Sigma}^{-1}\mathbf{1}_m &= \frac{1}{\sigma_y^2(1-\rho)}\left[\mathbf{1}_m^T\mathbf{1}_m - \frac{\rho\mathbf{1}_m^T\mathbf{1}_m\mathbf{1}_m^T\mathbf{1}_m}{1+(m-1)\rho}\right] \\
&= \frac{1}{\sigma_y^2(1-\rho)}\left[m - \frac{\rho m^2}{1+(m-1)\rho}\right] \\
&= \frac{m}{\sigma_y^2(1-\rho)}\left[1 - \frac{\rho m}{1+(m-1)\rho}\right] \\
&= \frac{m}{\sigma_y^2(1-\rho)}\frac{[1+(m-1)\rho-\rho m]}{[1+(m-1)\rho]} \\
&= \frac{m}{\sigma_y^2(1-\rho)}\frac{(1-\rho)}{[1+(m-1)\rho]} \\
&= \frac{m}{\sigma_y^2\left[1+(m-1)\rho\right]}.
\end{aligned}$$

We also can get

$$\begin{aligned}
\boldsymbol{u}_i\boldsymbol{u}_i^T &= \begin{pmatrix} 1 \\ x_i \end{pmatrix}(1, x_i) \\
&= \begin{pmatrix} 1 & x_i \\ x_i & x_i^2 \end{pmatrix}.
\end{aligned}$$

Hence, we have

$$\begin{aligned}
\boldsymbol{U}^T\boldsymbol{\Omega}^{-1}\boldsymbol{U} =& \mathbf{1}_m^T\boldsymbol{\Sigma}^{-1}\mathbf{1}_m \sum_{i=1}^{n} \boldsymbol{u}_i\boldsymbol{u}_i^T \\
=& \frac{m}{\sigma_y^2\left[1+(m-1)\rho\right]} \begin{pmatrix} n & \sum_{i=1}^{n} x_i \\ \sum_{i=1}^{n} x_i & \sum_{i=1}^{n} x_i^2 \end{pmatrix} \\
=& \frac{nm}{\sigma_y^2\left[1+(m-1)\rho\right]} \begin{pmatrix} 1 & \bar{x} \\ \bar{x} & \sum_{i=1}^{n} x_i^2/n \end{pmatrix}.
\end{aligned}$$

Note that based on the matrix theories, the inverse of a 2×2 matrix

$$\boldsymbol{A} = \begin{pmatrix} a & b \\ c & d \end{pmatrix}$$

is

$$\boldsymbol{A}^{-1} = \frac{\begin{pmatrix} d & -b \\ -c & a \end{pmatrix}}{ad-bc}.$$

Therefore,

$$\begin{aligned}
Cov\left(\hat{\boldsymbol{\beta}}\right) =& \left(\boldsymbol{U}^T\boldsymbol{\Omega}^{-1}\boldsymbol{U}\right)^{-1} \\
=& \frac{\sigma_y^2\left[1+(m-1)\rho\right]}{nm} \frac{1}{\left(\sum_{i=1}^{n} x_i^2/n - \bar{x}^2\right)} \begin{pmatrix} \sum_{i=1}^{n} x_i^2/n & -\bar{x} \\ -\bar{x} & 1 \end{pmatrix} \\
=& \frac{\sigma_y^2\left[1+(m-1)\rho\right]}{nm} \frac{1}{\frac{1}{n}\sum_{i=1}^{n}\left(x_i-\bar{x}\right)^2} \begin{pmatrix} \sum_{i=1}^{n} x_i^2/n & -\bar{x} \\ -\bar{x} & 1 \end{pmatrix} \\
=& \frac{\sigma_y^2\left[1+(m-1)\rho\right]}{m} \frac{1}{\sum_{i=1}^{n}\left(x_i-\bar{x}\right)^2} \begin{pmatrix} \sum_{i=1}^{n} x_i^2/n & -\bar{x} \\ -\bar{x} & 1 \end{pmatrix} \\
=& \frac{\sigma_y^2\left[1+(m-1)\rho\right]}{m(n-1)} \frac{1}{\hat{\sigma}_x^2} \begin{pmatrix} \sum_{i=1}^{n} x_i^2/n & -\bar{x} \\ -\bar{x} & 1 \end{pmatrix} \\
=& \frac{\left[1+(m-1)\rho\right]}{m(n-1)} \frac{\sigma_y^2}{\hat{\sigma}_x^2} \begin{pmatrix} \sum_{i=1}^{n} x_i^2/n & -\bar{x} \\ -\bar{x} & 1 \end{pmatrix},
\end{aligned} \tag{A12}$$

where

$$\hat{\sigma}_x^2 = \frac{1}{(n-1)} \sum_{i=1}^{n} \left(x_i - \bar{x}\right)^2.$$

Finally, we obtain

$$Var\left(\hat{\beta}_1\right) = \frac{[1+(m-1)\rho]}{m(n-1)}\frac{\sigma_y^2}{\hat{\sigma}_x^2}. \tag{A13}$$

Note that $Var\left(\hat{\beta}_1\right)$ is the variance conditional on x_1, …, x_n.

F Power calculation formula revisit

Based on Formulas (A10) and (A13), the power calculation formula for testing Hypotheses (33) can be rewritten as:

$$\begin{aligned} power =& 1-\Phi\left(z_{\alpha/2}-\frac{\delta}{\sqrt{Var\left(\hat{\beta}_1\right)}}\right)+\Phi\left(-z_{\alpha/2}-\frac{\delta}{\sqrt{Var\left(\hat{\beta}_1\right)}}\right) \\ =& 1-\Phi\left(z_{\alpha/2}-\frac{\hat{\sigma}_x}{\sigma_y}\frac{\delta\sqrt{m(n-1)}}{\sqrt{1+(m-1)\rho}}\right)+\Phi\left(-z_{\alpha/2}-\frac{\hat{\sigma}_x}{\sigma_y}\frac{\delta\sqrt{m(n-1)}}{\sqrt{1+(m-1)\rho}}\right), \end{aligned}$$

where α is the type I error rate, $z_{\alpha/2}$ is the upper $100\alpha/2$ percentile of the standard normal distribution $N(0,1)$, $\sigma_y = \sqrt{\sigma_\beta^2+\sigma^2}$, $\hat{\sigma}_x = \sqrt{\sum_{i=1}^n \left(x_i-\bar{x}\right)^2/(n-1)}$, and $\rho = \sigma_\beta^2/\left(\sigma_\beta^2+\sigma^2\right)$.

G Variance of genotype under Hardy-Weinberg Equilibrium

For the given SNP, suppose its minor allele frequency (MAF) is θ ($0<\theta<0.5$). Then under Hardy-Weinberg Equilibrium, the genotype frequencies are

$$\begin{aligned} Pr\left(x_i=2\right) =& \theta^2, \\ Pr\left(x_i=1\right) =& 2\theta\left(1-\theta\right), \\ Pr\left(x_i=0\right) =& \left(1-\theta\right)^2, \end{aligned}$$

The mean genotype is

$$\begin{aligned}E(x_i) =&2 \times Pr(x_i = 2) + 1 \times Pr(x_i = 1) + 0 \times Pr(x_i = 0)\\=&2\theta^2 + 2\theta(1-\theta)\\=&2\theta.\end{aligned}$$

We also can derive the second moment of genotype

$$\begin{aligned}E\left(x_i^2\right) =&2^2 \times Pr(x_i = 2) + 1^2 \times Pr(x_i = 1) + 0^2 \times Pr(x_i = 0)\\=&4\theta^2 + 2\theta(1-\theta)\\=&2\theta^2 + 2\theta\\=&2\theta(1+\theta).\end{aligned}$$

The variance of genotype is

$$\begin{aligned}Var\left(x_i^2\right) =&E\left(x_i^2\right) - \left[E(x_i)\right]^2\\=&2\theta(1+\theta) - 4\theta^2\\=&2\theta(1-\theta).\end{aligned}$$

H Power calculation formula for genotypes under Hardy-Weinberg Equilibrium

Hence, the power calculation formula for testing Hypotheses (33) for genotypes under Hardy-Weinberg Equilibrium is:

$$\begin{aligned}power =&1 - \Phi\left(z_{\alpha/2} - \frac{\sqrt{2\theta(1-\theta)}}{\sigma_y}\frac{\delta\sqrt{m(n-1)}}{\sqrt{1+(m-1)\rho}}\right)\\&+\Phi\left(-z_{\alpha/2} - \frac{\sqrt{2\theta(1-\theta)}}{\sigma_y}\frac{\delta\sqrt{m(n-1)}}{\sqrt{1+(m-1)\rho}}\right).\end{aligned}$$

where α is the type I error rate, $z_{\alpha/2}$ is the upper $100\alpha/2$ percentile of the standard normal distribution $N(0,1)$, $\sigma_y = \sqrt{\sigma_\beta^2 + \sigma^2}$, θ is the minor allele frequency, and $\rho = \sigma_\beta^2/\left(\sigma_\beta^2 + \sigma^2\right)$ is the intra-class correlation.

References

[1] Dupont, W.D. and Plummer, W.D. (1998). Power and sample size calculations for studies involving linear regression. *Controlled Clinical Trials*, 19:589–601.

[2] O'Brien, RG and Muller, KE (1993). Unified power analysis for t-tests through multivariate hypotheses. In Edwards, L., editor, *Applied Analysis of Variance in Behavioral Science*, pages 297–344. New York: Dekker.

[3] The GTEx Consortium (2013). The genotype-tissue expression (GTEx) project. *Nature Genetics*, 45:580–585.

[4] Vieth, B, Ziegenhain, C, Parekh, S, Enard, W, and Hellmann, I (2017). powsimR: power analysis for bulk and single cell rna-seq experiments. *Bioinformatics*, 33(21):3486–3488.

[5] Zhang Z, Yu D, Seo M, Hersh CP, Weiss ST, and Qiu W (2019a). *countTransformers: Transform Counts in RNA-Seq Data Analysis*. R package version 0.0.6.

[6] Zhang Z, Yu D, Seo M, Hersh CP, Weiss ST, and Qiu W (2019b). Novel data transformations for RNA-seq differential expression analysis. *Scientific Reports*, 9:4820.

[7] Zheng, FH (2016). The design and statistical analysis of single-cell rna-sequencing experiments. *Open Access Dissertations*, (897).
